# Supplementary material for: Single-molecule diffusivity quantification in Xenopus egg extracts elucidates physicochemical properties of the cytoplasm
Source: Proc Natl Acad Sci U S A. 2024 Dec 5;121(50):e2411402121. doi: 10.1073/pnas.2411402121 (PMC11648914; doi:10.1073/pnas.2411402121)
Supplement: Supplementary file 1 — Appendix 01 (PDF) [file pnas.2411402121.sapp.pdf]

Table S1. Diffuser proteins used in this work, their Uniprot IDs, molecular weights (MWs), estimated net charges, and sources. Net charges are estimated using the canonical "Chain" sequences (signal peptides removed) according to Uniprot. Charge estimation is performed with Prot pi (https://www.protpi.ch/) for pH 7.7 (typical pH of the *Xenopus* egg cytoplasm and extract (*1*)) using the ExPASy isoelectric point values, with disulfide bonds applied globally. A shift of −1 net charge is assessed for samples labeled by Cy3B NHS ester. Whereas Cy3B NHS ester is charge-neutral, its conjugation to lysine removes one positive charge on the labeled protein and so shifts the net charge by −1.

Label in figures	Protein	Uniprot ID	MW (kDa)	Charge @pH7.7	+Cy3B MW(kDa)	+Cy3B Charge	Provider	Cat#
**Apoferritin**	Apoferritin, equine (24-mer)	P02791 Q8MIP0	480	-235	480	-236	Sigma	A3660
**THYG**	Thyroglobulin, bovine (dimer)	P01267	660	-83	660	-84	Sigma	T1001
**BMY1**	Beta amylase, sweet potato (tetramer)	P10537	224	-59	224	-60	Sigma	A8781
**ADH**	Alcohol dehydrogenase, yeast (tetramer)	P00330	150	-19.4	151	-20.4	Sigma	A8656
**BSA**	Bovine serum albumin	P02769	66.4	-17.7	67	-18.7	Sigma	A3059
**STI**	Soybean trypsin inhibitor	P01070	20.0	-11.6	20.6	-12.6	Sigma	T2327
**BCA**	Bovine carbonic anhydrase	P00921	29.1	-3.8	29.7	-4.8	Sigma	C2624
**sHEWL***	Succinylated HEWL	-	14.8	-2.6	15.4	-3.6	From HEWL	From HEWL
**CTRA**	Chymotrypsinogen A, bovine	P00766	25.7	3.3	26.3	2.3	Sigma	C4879
**RNase A**	RNase A, bovine	P61823	13.7	3.4	14.3	2.4	Sigma	R5500
**HEWL**	Lysozyme from chicken egg white	P00698	14.3	7.4	14.9	6.4	Sigma	L4919
**Avidin**	Avidin from chicken egg white (tetramer)	P02701	68	21.6	68.6	20.6	Sigma	A9275
**Cas9-NLS**[#]	Cas9, recombinant, with 1X NLS	Q99ZW2	160	24.0	161	23.0	Sigma	CAS9PROT
**H1.0**	Histone H1.0, human, recombinant	P07305	20.7	52	21.3	51	NEB	M2501S
**PolyK**	poly(D,L-lysine hydrobromide)$_{250}$	-	52	250	52.6	250	Alamanda Polymers	000-RKB250

*sHEWL: Assuming Cy3B NHS ester conjugated to one of the 6 lysine residues, while succinylation of the remaining 5 lysine residues inversed five +1 charges to −1 (*2*). This still leaves one N-terminus amine that may be succinylated.

[#]Cas9-NLS: Based on the sequence of Cas9 plus the +5 charged typical nuclear localization sequence (NLS) PKKKRKV.

Table S2. List of the most abundant cytoplasmic proteins in the *Xenopus* egg cytoplasmic extract and their estimated net charge *q*. Cytoplasmic proteins (not including RNA-binding and ribosomal proteins in Table S3) are ordered by their estimated concentrations *c* in the extract according to the mass spectrometry data of (*3*), based on updated annotations by Wühr Lab (https://wuehr.scholar.princeton.edu/protein-concentrations-xenopus-egg). Net charges at pH 7.7 are estimated for the annotated sequences as described in Table S1. Most proteins on this list are negatively charged or neutral. A few proteins in the glycolysis pathway (GAPDH and ALDOA are weakly positively charged (+1 to +3), which may mediate binding to negatively charged phosphorylated glucose metabolites, thereby neutralizing the total charge.

***Laevis* gene**	**Human gene**	**Protein description**	***c* (µM)**	**Net *q***
serpina6.L	SERPINA1	Alpha-1-antitrypsin	8.7	0.2
actc1.L	ACTC1	Actin, alpha cardiac muscle 1	8.0	-13.8
eno1.L	ENO1	Alpha-enolase	7.9	-6.9
cgl.2.L	ITLN2	Intelectin-2	6.3	-7.7
hbz.L	HBZ	Hemoglobin subunit zeta	5.9	-4.8
actg1.S	ACTG1	Actin, cytoplasmic 2	5.8	-12.8
gapdh.L	GAPDH	Glyceraldehyde-3-phosphate dehydrogenase	5.8	2.1
tpi1.S	TPI1	Triosephosphate isomerase	5.8	-0.9
ldhb.S	LDHB	L-lactate dehydrogenase B chain	5.5	-6.6
nme2.S	NME2	Nucleoside diphosphate kinase B	5.3	-1.9
fabp4	FABP4	Fatty acid-binding protein, adipocyte	4.6	-1.9
rps27a.S*	RPS27A	Ubiquitin	4.4	-0.9
pkm.L	PKM	Pyruvate kinase PKM	4.2	-8.8
plin2.L	PLIN2	Perilipin-2	4.2	-16.9
grhpr.2.L	GRHPR	Glyoxylate reductase/hydroxypyruvate reductase	4.1	-4.9
nme2.L	NME2	Nucleoside diphosphate kinase B	4.1	-1.9
mdh1.S	MDH1	Malate dehydrogenase, cytoplasmic	4.1	-1.9
hba-l5.L	HBZ	Hemoglobin subunit zeta	4.0	-4.8
ckb.S	CKB	Creatine kinase B-type	3.9	-7.8
pgk1.L	PGK1	Phosphoglycerate kinase 1	3.9	-1.9
gapdh.S	GAPDH	Glyceraldehyde-3-phosphate dehydrogenase	3.9	1.1
aldoa.S	ALDOA	Fructose-bisphosphate aldolase A	3.7	3.2
gstm1.S	GSTM1	Glutathione S-transferase Mu 1	3.7	0.1
hpgds.S	HPGDS	Hematopoietic prostaglandin D synthase	3.6	-2.0
ahcy.S	AHCY	Adenosylhomocysteinase	3.6	-6.9
ftmt.S	FTH1	Ferritin heavy chain	3.5	-11.7
gpi.L	GPI	Glucose-6-phosphate isomerase	3.4	-2.7
tpi1.L	TPI1	Triosephosphate isomerase	3.3	-1.9

* rps27a.S (RPS27A) encodes both ubiquitin and the 40S ribosomal protein S27a. The charge here is calculated for ubiquitin.

Table S3. List of the most abundant RNA-binding and ribosomal proteins in the *Xenopus* egg cytoplasmic extract as determined by mass spectrometry (*3*), and their estimated net charges, generated as in Table S2. Note that when assembled with rRNA into ribosomes, the strong negative charges on rRNA (−1 net charge per nucleotide on the RNA backbone) overcompensate for protein charges to render the assembled ribosomes highly negatively charged (*4, 5*).

***Laevis* gene**	**Human gene**	**Protein description**	***c* (µM)**	**Net *q***
rpl32.L	RPL32	60S ribosomal protein L32	5.0	25.1
rpl23a.S	RPL23A	60S ribosomal protein L23a (Fragment)	4.7	25.1
rps27a.S*	RPS27A	40S ribosomal protein S27a	4.4	15.4
cirbp.S	CIRBP	Cold-inducible RNA-binding protein	4.1	2.1
rps14.L	RPS14	40S ribosomal protein S14	4.1	12.2
rps9	RPS9	40S ribosomal protein S9	4.0	21.1
rpl27.S	RPL27	60S ribosomal protein L27	3.9	24.0
rpl7a.S	RPL7A	60S ribosomal protein L7a	3.8	40.0
rps18	RPS18	40S ribosomal protein S18	3.8	33.1
rps16.S	RPS16	40S ribosomal protein S16	3.8	16.1
rps3.L	RPS3	40S ribosomal protein S3	3.7	11.1
rps8.L	RPS8	40S ribosomal protein S8	3.7	33.1
rps28p9.L	RPS28	40S ribosomal protein S28	3.6	4.2
LOC398653	RPLP2	60S acidic ribosomal protein P2	3.5	-8.9
rplp0.S	RPLP0	60S acidic ribosomal protein P0	3.5	-6.9
rps4x.L	RPS4X	40S ribosomal protein S4, X isoform	3.4	23.2
rps12.L	RPS12	40S ribosomal protein S12	3.4	-0.9
rpl27a.S	RPL27A	60S ribosomal protein L27a	3.4	22.3
rpl26.L	RPL26	60S ribosomal protein L26	3.3	24.1
rpl22.L	RPL22	60S ribosomal protein L22	3.3	6.1
rpl8.L	RPL8	60S ribosomal protein L8	3.3	36.2
rps19.S	RPS19	40S ribosomal protein S19	3.3	16.2
rps17.S	RPS17	40S ribosomal protein S17	3.3	8.1
rps25.L	RPS25	40S ribosomal protein S25	3.3	20.0
rps11	RPS11	40S ribosomal protein S11	3.1	22.1
rpl19	RPL19	60S ribosomal protein L19	3.1	43.1
rpl7.L	RPL7	60S ribosomal protein L7	3.1	41.0
rps6.L	RPS6	40S ribosomal protein S6	3.0	40.0
rpl4.S	RPL4	60S ribosomal protein L4	3.0	63.0

* rps27a.S (RPS27A) encodes both ubiquitin and the 40S ribosomal protein S27a. The charge here is calculated for the 40S ribosomal protein S27a.

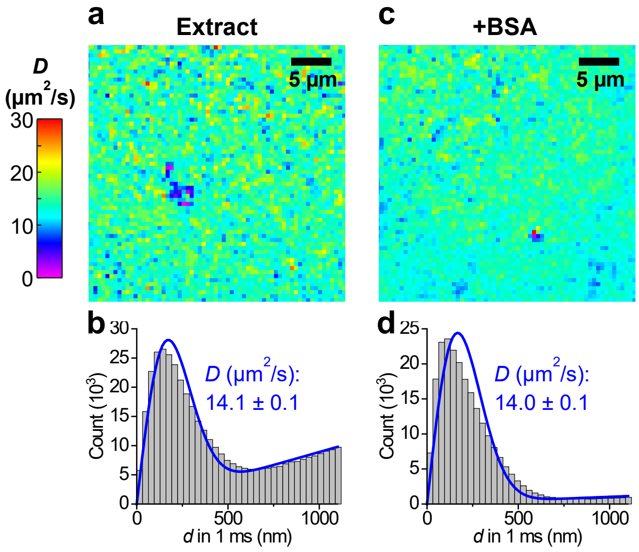

Figure S1. SM*d*M diffusivity mapping of Cy3B-labeled HEWL in untreated and BSA-supplemented *Xenopus* egg extracts. (**a**) Color-coded SM*d*M diffusivity map for an untreated sample, presented on the same color scale as **Fig. 3**. (**b**) Distribution of the 1-ms single-molecule displacements from (a). Blue curve: fit to our single-mode SM*d*M diffusion model, with resultant apparent diffusion coefficients D and 95% confidence intervals marked. Note that in this sample, a higher concentration of Cy3B-labeled HEWL (~1 nM) was used to increase the detected single-molecule density to facilitate spatial mapping. While this led to a higher background in the displacement distribution (when compared to **Fig. 1d**), the D value obtained with our fitting model (Eqn. 1) stays consistent, in agreement with what we have analyzed and demonstrated previously (*6*). (**c**) Color-coded SM*d*M diffusivity map for another sample to which BSA was added at 1 mg/mL. (**d**) Distribution of the 1-ms single-molecule displacements from (c) and fit to our model.

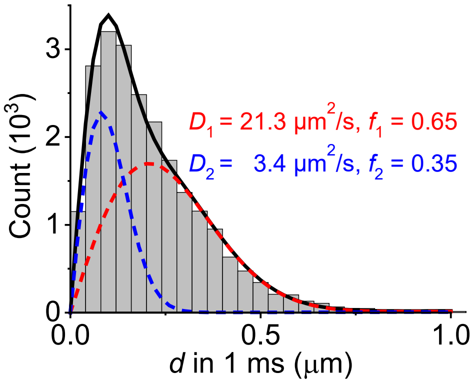

Figure S2. Two-component D fit to the distribution of SM*d*M-recorded 1-ms single-molecule displacements of Cy3B-labeled HEWL diffusing in extract as shown in **Fig. 1d**. Histogram: displacements; Red curve: fast component of the fit (D_1 = 21.3 μm²/s, fraction f_1 = 0.65); Blue curve: slow component of the fit (D_2 = 3.4 μm²/s, fraction f_2 = 0.35); Black curve: sum of the red and blue curves. While the two-component fit improves over the single-component model (**Fig. 1d**), the system may be better assumed as a continuous distribution of different transient states.

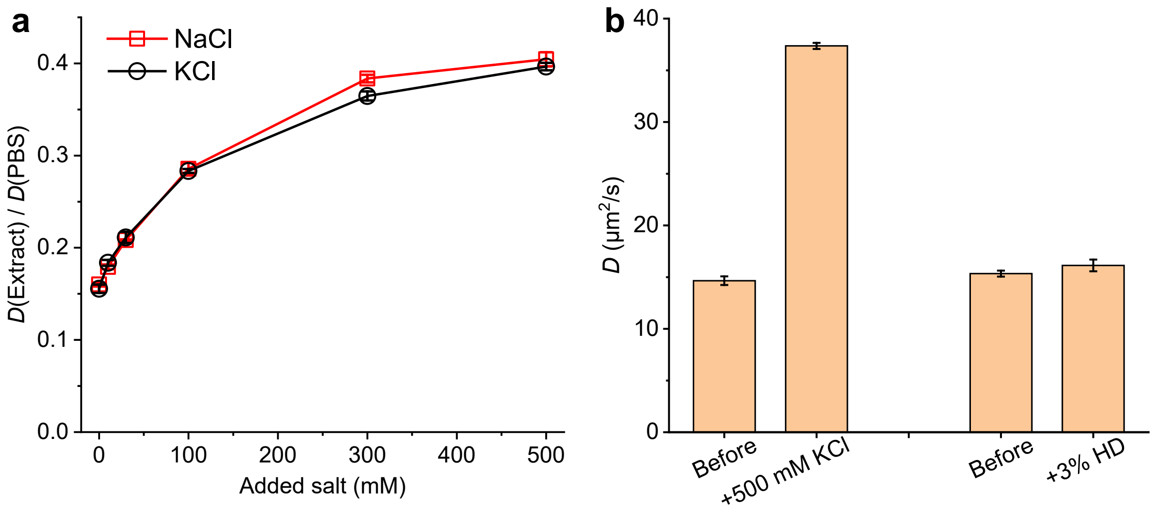

Figure S3. Diffusion of Cy3B-labeled HEWL in extracts supplemented with NaCl, KCl, and 1,6-hexanediol. (a) SM*d*M-determined relative in-extract *D* values of Cy3B-labeled HEWL normalized to in-PBS values, for two samples separately added with NaCl (red) and KCl (black) to different concentrations. Error bars: Sample standard deviations between results from three SM*d*M measurements at each data point. (b) SM*d*M-determined *D* values of Cy3B-labeled HEWL in two extract samples, before and after separately adding 500 mM KCl or 3% 1,6-hexanediol. Error bars: Sample standard deviations between results from three SM*d*M measurements.

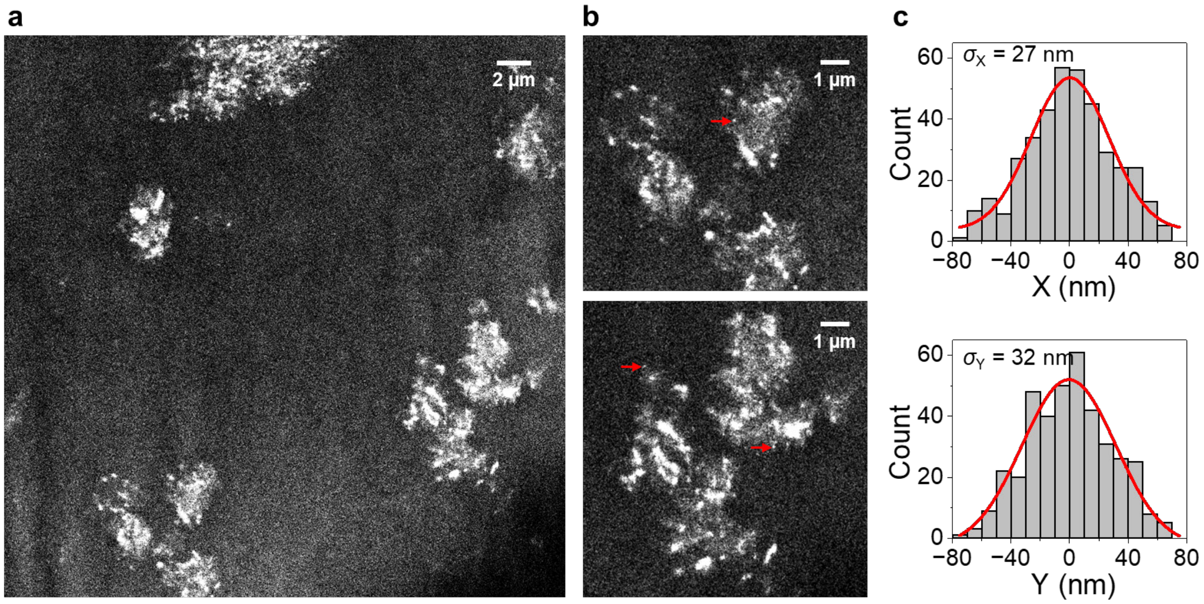

Figure S4. SMLM super-resolution images of Cy3B-labeled HEWL in RNase-treated extract, as generated from the single-molecule localizations of the SM*d*M data. (a) Same field of view as the SM*d*M data of Fig. 3c. (b) Zoom-ins of two regions. (c) Distribution of single-molecule positions for overlaid 4 small nanoclusters like those indicated by the red arrows in (b), in the X (top) and Y (bottom) directions, respectively. Gaussian fits (red curves) give standard deviations of 27 and 32 nm.

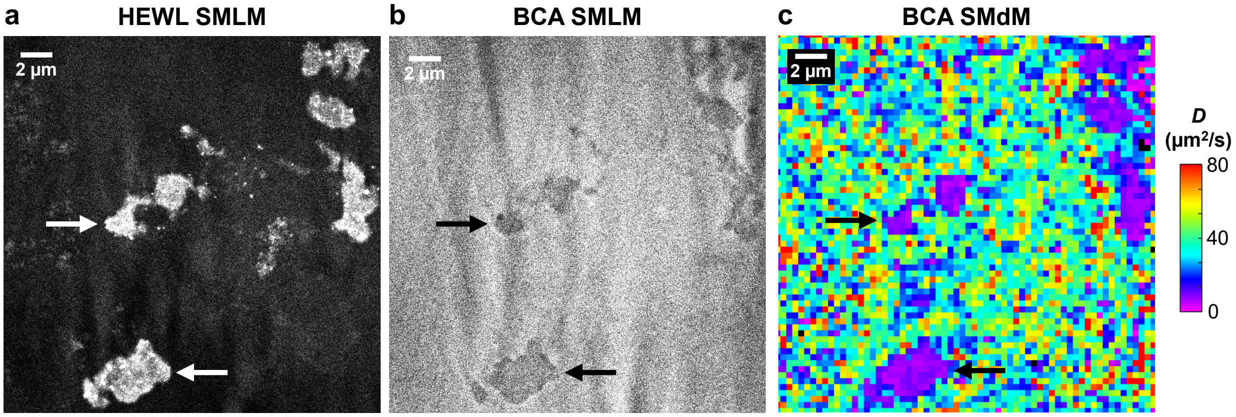

Figure S5. Diffusion of the negatively charged BCA in RNase-treated extract. (a,b) SMLM images of Cy3B-labeled HEWL (a) and CF647-labeled BCA (b) in an RNase-treated extract sample. Vertical stripe patterns are attributed to local lensing effects from the high refractive indices of the aggregates under our inclined illumination scheme (*7*). (c) SM*d*M diffusivity map of the CF647-labeled BCA. Arrows point to aggregates, where diffusivity reduction was accompanied by local increases and decreases in the abundances of HEWL and BCA, respectively. These results may be interpreted as that as the RNase-released positively charged proteins interact with the negatively charged cytoplasm environment, the resultant aggregates are overwhelmed by the latter on the surface. Positively and negatively charged tracer proteins are thus respectively attracted to and repelled by the aggregates, with the former further likely participating in the aggregate cores.

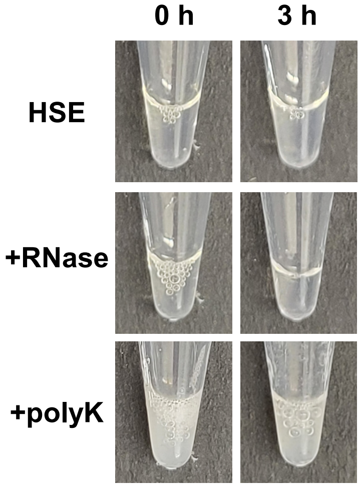

Figure S6. Aggregation assays for the ribosome-depleted high-speed extracts (HSEs). Photos are shown for HSEs at 0 h (left) and 3 h (right), without or with the addition of RNase or 1 mg/mL polylysine. Whereas polylysine addition generated immediate clouding, RNase treatment did not induce clouding over 3 h, consistent with our model that in the crude, ribosome-containing cytoplasm extract, RNase degradation of rRNA releases positively charged ribosomal proteins to cause aggregation.

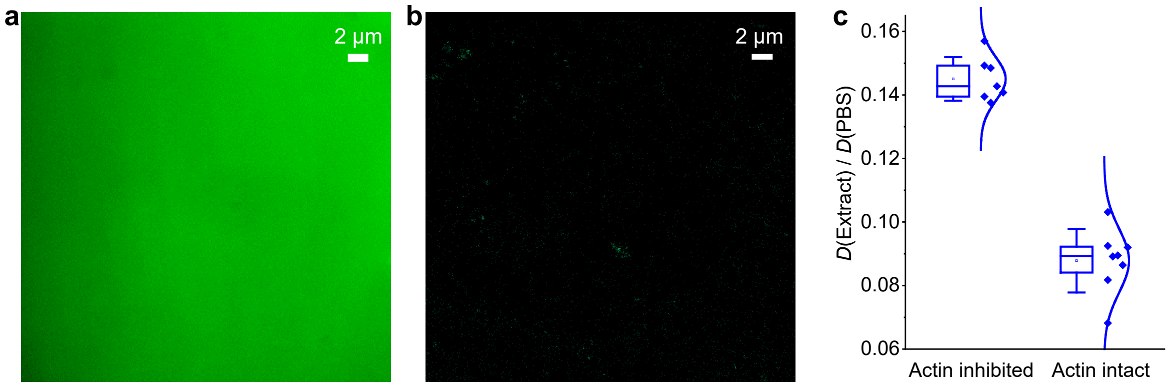

Figure S7. Additional data related to actin filaments. (a,b) Typical epifluorescence (a) and STORM (b) images of phalloidin-AF647 in actin-inhibited *Xenopus* egg extracts, showing no resolvable structures. (c) SM*d*M-determined *D* values in the extract relative to in PBS, for the positively charged HEWL in actin-inhibited and actin-intact extracts. Each data point corresponds to one independent SM*d*M measurement for a different sample region, from 5 actin-inhibited and 3 actin-intact samples, respectively.

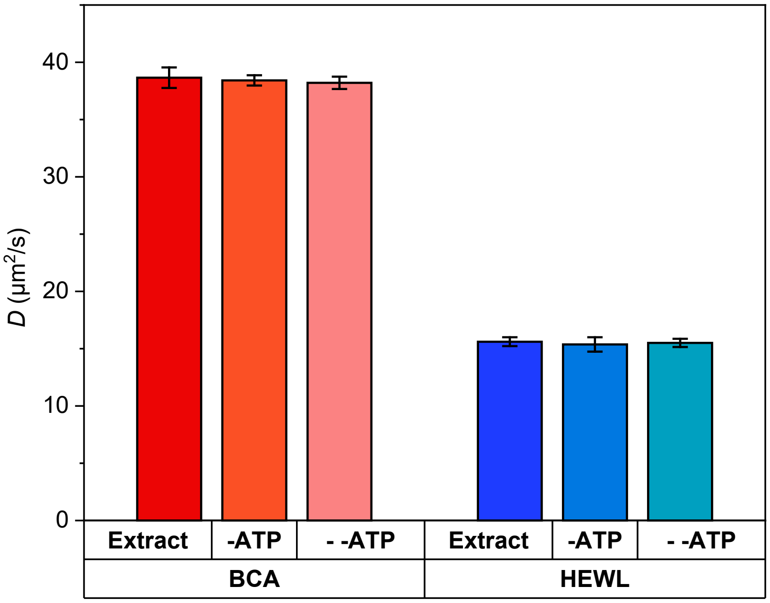

Figure S8. SM*d*M-determined *D* values of CF647-labeled BCA and Cy3B-labeled HEWL in extracts with different ATP levels. "Extract": Typical samples added with 1x energy mix (4 mM creatine phosphate, 0.5 mM ATP, 0.05 mM EGTA, and 0.5 mM $MgCl_2$). "-ATP": Samples obtained in the same preparation but without addition of the energy mix. "- -ATP": "-ATP" samples further subjected to ATP depletion by apyrase [New England Biolabs M0398S; incubated at 1:40 (12.5 units/mL) for 20 min], a condition known to abolish energy-dependent processes in extract, *e.g.*, cell-like compartmentalization (*8*) and DNA compaction (*9*). Error bars: Sample standard deviations between results from three SM*d*M measurements.

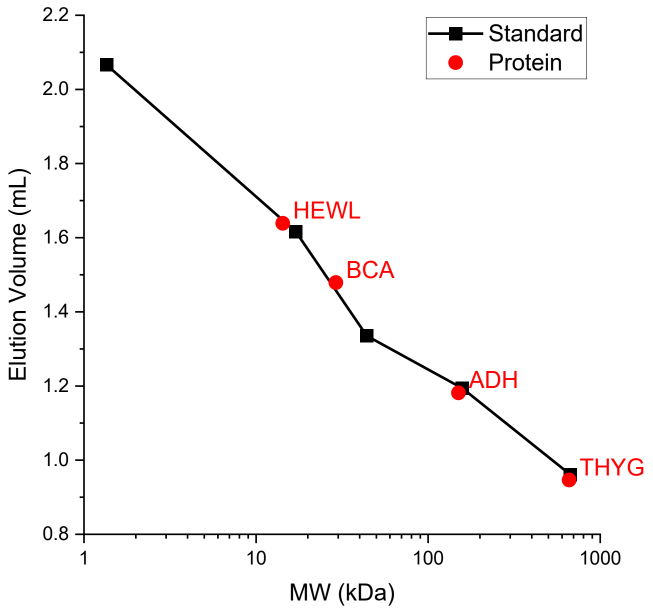

Figure S9. Size-exclusion chromatography elution volumes of four of the protein samples used in this study (red circles) compared to calibration standards (Bio-Rad 1511901; black squares), plotted versus the expected molecular weight. Separation was performed at 4 °C in PBS. 50 µL of protein solution (1 mg/mL) was injected into an ÄKTA pure micro chromatography system (Cytiva 29302479) equipped with a Superdex 200 Increase 3.2/300 column (Cytiva 28990946) at a flow rate of 0.025 mL/min. Sample elution was monitored by absorbance at 280 nm.

References for Supplement

1. M. C. Good, R. Heald, Preparation of cellular extracts from *Xenopus* eggs and embryos. *Cold Spring Harb. Protoc.*, doi:10.1101/pdb.prot097055 (2018).
2. I. Gitlin, J. D. Carbeck, G. M. Whitesides, Why are proteins charged? Networks of charge-charge interactions in proteins measured by charge ladders and capillary electrophoresis. *Angew. Chem. Int. Ed.* **45**, 3022-3060 (2006).
3. M. Wühr, R. M. Freeman, M. Presler, M. E. Horb, L. Peshkin, S. P. Gygi, M. W. Kirschner, Deep proteomics of the *Xenopus laevis* egg using an mRNA-derived reference database. *Curr. Biol.* **24**, 1467-1475 (2014).
4. A. M. Knight, P. H. Culviner, N. Kurt-Yilmaz, T. S. Zou, S. B. Ozkan, S. Cavagnero, Electrostatic effect of the ribosomal surface on nascent polypeptide dynamics. *ACS Chem. Biol.* **8**, 1195-1204 (2013).
5. P. E. Schavemaker, W. M. Smigiel, B. Poolman, Ribosome surface properties may impose limits on the nature of the cytoplasmic proteome. *eLife* **6**, e30084 (2017).
6. L. Xiang, K. Chen, R. Yan, W. Li, K. Xu, Single-molecule displacement mapping unveils nanoscale heterogeneities in intracellular diffusivity. *Nat. Methods* **17**, 524-530 (2020).
7. C. He, C. Y. Wu, W. Li, K. Xu, Multidimensional super-resolution microscopy unveils nanoscale surface aggregates in the aging of FUS condensates. *J. Am. Chem. Soc.* **145**, 24240-24248 (2023).
8. X. R. Cheng, J. E. Ferrell, Spontaneous emergence of cell-like organization in *Xenopus* egg extracts. *Science* **366**, 631-637 (2019).
9. M. Sun, H. Amiri, A. B. Tong, K. Shintomi, T. Hirano, C. Bustamante, R. Heald, Monitoring the compaction of single DNA molecules in *Xenopus* egg extract in real time. *Proc Natl Acad Sci U S A* **120**, e2221309120 (2023).
